# Shared Genetic Architecture of Premenstrual Disorder and Postpartum Depression: Registry-Based and Genetic Evidence

**DOI:** 10.21203/rs.3.rs-10380518/v1

**Published:** 2026-08-07

**Authors:** Susu Qu, Kejia Hu, Jerry Guintivano, Elgeta Hysaj, Piotr Jaholkowski, Alexey A. Shadrin, Alicia Nevriana, Rebecka Keijser, Yi Lu, Joeri Meijsen, Bowen Tang, Unnur A. Valdimarsdóttir, Alkistis Skalkidou, Sarah A. Rudzinskas, Triin Laisk, David Goldman, Peter J. Schmidt, Ole A. Andreassen, Donghao Lu

**Affiliations:** 1 Unit of Integrative Epidemiology, Institute of Environmental Medicine, Karolinska Institutet, Stockholm, Sweden; 2 Sleep Medicine Center, Mental Health Center, National Center for Mental Disorders, Sleep Research Laboratory, West China Hospital, Sichuan University, Chengdu, China; 3 West China Biomedical Big Data Center, West China Hospital, Sichuan University, Chengdu, China; 4 Department of Psychiatry, University of North Carolina at Chapel Hill, Chapel Hill, NC, USA; 5 Department of Genetics, University of North Carolina at Chapel Hill, Chapel Hill, NC, USA; 6 Center for Precision Psychiatry, Division of Mental Health and Addiction, Oslo University Hospital, and Institute of Clinical Medicine, University of Oslo, Oslo, Norway; 7 KG Jebsen Centre for Neurodevelopmental Disorders, University of Oslo and Oslo University Hospital, Oslo, Norway; 8 Department of Oncology and Pathology, Karolinska Institutet, Stockholm, Sweden; 9 Department of Medical Epidemiology and Biostatistics, Karolinska Institutet, Stockholm, Sweden; 10 Institute of Biological Psychiatry, Mental Health Center Sct. Hans, Amager & Hvidovre Hospital, Copenhagen University Hospital, Mental Health Services Copenhagen, Roskilde, Denmark; 11 Center of Public Health Sciences, Faculty of Medicine, University of Iceland, Reykjavík, Iceland; 12 Department of Epidemiology, Harvard T.H. Chan School of Public Health, Boston, Massachusetts, USA; 13 Department of Women’s and Children’s Health, Uppsala Universitet, Uppsala, Sweden; 14 Behavioral Endocrinology Branch, NIMH, Bldg. 10CRC, Room 25330, 10 Center Drive MSC 1277, Bethesda 20892-1277 MD, USA; 15 Office of the Clinical Director and Laboratory of Neurogenetics, NIAAA, Bethesda, MD, USA; 16 Estonian Genome Centre, Institute of Genomics, University of Tartu, Tartu, Estonia

**Keywords:** Postpartum depression, premenstrual syndrome, premenstrual dysphoric disorder, genetic correlation, heritability, GABA_B_ signaling

## Abstract

**Background:**

Premenstrual disorder (PMD) and postpartum depression (PPD) have a strong phenotypic link and echo women’s hormone fluctuations. Yet, the extent to which they may be cross-inherited remains poorly understood.

**Methods:**

Using the nationwide cohort of 907,841 women who born 1950-2007 and gave birth during 2001-2021 in Sweden, we estimated the cumulative incidence functions-based heritability and genetic correlation for PMD and PPD. We also analyzed genome-wide association study (GWAS) summary statistics from the largest European-ancestry cohorts for PMD (17,511 cases and 54,786 controls) and PPD (16,145 cases and 46,609 controls) using linkage disequilibrium score regression (LDSC). Fixed-effect cross-trait meta-analysis and imputed transcriptome-wide association analyses (TWAS) were conducted to identify shared loci and gene-tissue associations.

**Results:**

The register-based heritability was 0.35 (95% CI: 0.29-0.41) for PMD and 0.31 (95% CI: 0.23-0.37) for PPD, with a positive genetic correlation between these disorders (r_g_ = 0.47, 95% CI: 0.25–0.69). LDSC also showed a positive genetic correlation between PMD and PPD (*r*_g_ = 0.66, SE = 0.10, *P* = 1.014×10^−10^), indicating sizable shared heritable influences. Cross-trait meta-analysis identified two novel genome-wide significant loci jointly associated with PMD and PPD, mapping to an intronic region of *PCDH9* and the 3’ untranslated region of *KCTD16*. The *KCTD16* locus implicates GABA_B_–mediated inhibitory signaling in both disorders. Consistent with this, TWAS revealed a hippocampus regulatory signal for *KCTD16*, with no detectable trend effects in other brain regions or peripheral tissues. Beyond the lead loci, TWAS suggested that the shared risk variants may partially act through genetically regulated gene expression across brain, endocrine and immune-related tissues.

**Conclusions:**

Together, these findings provide the first evidence for sizable genetic overlap between PMD and PPD and highlight novel and convergent biological mechanisms underlying the abnormal brain response of some women to gonadal hormone fluctuations.

## Background

Reproductive endocrine-related mood disorders, such as premenstrual disorder (PMD) and postpartum depression (PPD), are characterized by affective symptoms during distinct reproductive phases in tandem with hormone fluctuations [1]. PMD typically emerges during the luteal phase of the menstrual cycle, while PPD usually occurs in the first months after childbirth. Both disorders affect millions of women worldwide, with an estimated prevalence of 20-30% for PMD and 17% of new mothers experiencing PPD [2, 3]. Prospective studies including our own [4, 5], have demonstrated a strong phenotypic link between PMD and PPD, highlighting a potential common underlying biologic susceptibility. The evidence is compelling that both PMD and PPD, are triggered by an abnormal response to changes in levels of ovarian steroids that naturally vary within women with menstrual cycle and due to pregnancy and parturition[6, 7]. The biological mechanisms remain largely elusive [8, 9], limiting treatment options for both conditions.

Emerging evidence suggests that genetic factors predispose some women to affective illness in response to normal hormone fluctuations [8, 9]. Twin studies have demonstrated moderate to high heritability for both conditions, with estimates of 31-56% for PMD [10] and 54% for PPD [11]. Moreover, a large-scale genome-wide association study (GWAS) meta-analysis of PPD (18,770 cases and 58,461 controls) has demonstrated moderate single nucleotide polymorphism (SNP)-based heritability (*h^2^SNP* = 0.14), though no robust genome-wide significant loci were found [12]. Recently, our group conducted the first and the largest genome-wide association study (GWAS) of PMD, comprising 17,511 cases and 54,786 controls of European ancestry, and identifying one genome-wide significant locus (*h^2^SNP* = 0.072) [13]. The findings of moderate to high heritability but few loci of large effect are congruent with, and further support, the complex polygenic nature of both disorders. Identifying shared genetic risk loci and biological pathways may inform future mechanistic research and facilitate the development of novel diagnostics and treatments.

In this study, we used Swedish nationwide registry data on all women born 1950-2007 to estimate cumulative incidence functions (CIF)-based heritability and genetic correlation, an approach developed to account for differences in age-at-onset and secular trends by stratifying on birth year [14]. We further analyzed the largest GWAS summary statistics of PMD and PPD to systematically investigate their shared genetic architecture. By integrating complementary cross-trait genomic and functional approaches, including cross-trait enrichment analyses and transcriptome-wide association studies (TWAS), we aimed to identify shared risk loci, and gain insight into the biological pathways underlying the affective susceptibility of some women to gonadal hormone fluctuations.

## Methods

### Nationwide register-based genetic-informed analysis in Sweden

We conducted a nationwide register-based familial analysis for all women who were born between 1950-2007, and gave birth during reproductive age (15–51 years) between 2001 and 2022, identified through the Total Population Register (TPR) [15]. For the present analyses of PMD and PPD, we restricted the study population to parous women with at least one recorded childbirth during 2001-2022 in Medical Birth Register to ensure comparable reproductive exposure.

As described elsewhere [16], PMD diagnoses were ascertained from the National Patient Register (NPR, specialist care) and Primary Care Register (PCR) using ICD-9 code 625E and ICD-10 code N94.3. NPR contains information of all inpatient stays from 1987 and specialized outpatient care since 2001 [17]. PCR includes primary care visits from Stockholm (from 2001), Skåne (from 2001), Uppsala (from 2005), Västra Götaland (2003-) and Värmland (from 2014). These counties cover 58–62% of women of reproductive age from 2001 to 2022 in Sweden. Prescribed Drug Register (PDR) includes data on prescribed drugs dispensed at pharmacies and was established with its current form from 2005. PDR was used to search for filled antidepressants (ATC code: N06A) or hormonal contraceptives (ATC code: G02B, G03A) based on recorded specific indication for PMDs as a complement to counties where PCR data were not available.

PPD was defined as a depressive episode occurring within 12 months after childbirth, based on specialist or primary care diagnoses (ICD-10 codes F32, F33, and F53.0), and antidepressant dispensation recorded in the PDR (ATC code: N06A) [18]. Full sister pairs were identified through the Multi-Generation Register (MGR) [19]. MGR includes data on family relationships for individuals born from 1932 and onwards [19].

Heritability (*h*^2^) was estimated under the liability threshold model using cumulative incidence functions (CIFs) stratified by birth year, comparing population CIF with within-trait CIF in full sisters (CIF of the same trait among women with a diagnosed sister) [14]. Genetic correlation (*r*_g_) between PMD and PPD was derived from cross-trait CIF (CIF of PMD among women with a sister diagnosed with PPD) and previously estimated *h*^2^ values, following the approach described before [20, 21]. Birth-year–specific estimates were meta-analyzed using random-effects inverse variance weighting.

This study was approved by the Swedish Ethical Review Authority (2021-02775 and 2025-02016-01). Informed consent was waived for register-based studies according to Swedish legislation.

### GWAS cohorts and data

We used summary statistics from the first GWAS of PMD in Nordic population, which included 17,511 PMD cases and 54,786 controls of European ancestry[13]. Participants comprised women from two Nordic cohorts - the Swedish LifeGene study (with 1,962 cases and 3,267 controls) and the Norwegian Mother, Father and Child Cohort Study (MoBa) (with 15,549 cases and 51,519 controls). The LifeGene cohort (started in 2009 with longitudinal follow-ups) enrolled 24,265 women aged 18-50 years at baseline, from whom blood samples were collected [22]. The MoBa cohort recruited participants from 1999 to 2008, including approximately 95,200 mothers [23, 24]. Cases with PMD were identified through either clinical registers or self-reported questionnaires. Specifically, questionnaires-based case definitions in LifeGene relied on a modified Premenstrual Symptom Screening Tool (PSST) [25, 26], while MoBa used screening questions at the 15^th^ week of gestation to recall symptom timing before pregnancy[27]. Controls were defined as women without clinical diagnoses of PMD or questionnaire evidence of meeting the PMD criteria from any available questionnaire cycles.

For PPD, we used a non-overlapping PPD GWAS dataset provided by collaborators, comprising 16,145 PPD women cases and 46,609 women controls of European ancestry. All samples overlapping with the PMD cohort were removed, and the final dataset included participants drawn from 15 European ancestry cohorts reported by Guintivano et al [12], with the MoBa cohort excluded. Cases were defined as individuals with a lifetime diagnosis of PPD occurring within one year after childbirth, identified through electronic medical records, the Edinburgh Postnatal Depression Scale (EPDS) [28], structured clinical interviews, or other self-report. Medical record-based diagnoses adhered to standard classification systems (DSM-IV, ICD-9, or ICD-10). The EPDS-based identification used a 10-item self-report tool focused on current symptoms; a modified version can screen for a lifetime history of PPD [29]. A cutoff score of ≥ 13 was applied to define cases [30]. Controls were required to have no lifetime history of MDD and at least one live, full-term birth (≥ 36 weeks’ gestation).

The initial datasets contained genotyped and imputed SNPs ranging from 6.98 to 9.75 million SNPs. We included only common SNPs (with minor allele frequency, MAF > 0.01) that were present in both datasets (PMD and PPD trait). All studies were approved by ethics committees, and participants gave written informed consent. For more details regarding the cohort and diagnosis information, please refer to the original study [12, 13].

### Global genetic correlation

We estimated SNP-based genetic correlations (*r*_g_) between PMD and PPD using linkage disequilibrium (LD) score regression (LDSC, Version 2.0.0) [31, 32], which has been demonstrated robust to both LD structure and sample overlap among summary-data based methods [33]. The analysis employed the HapMap 3 SNP reference panel, with linkage disequilibrium (LD) scores derived from European-ancestry individuals in the 1000 Genomes Project.

### Polygenic overlap quantification by MiXeR analysis

We quantified the polygenic overlap between PMD and PPD using a bivariate Gaussian mixture model implemented in MiXeR v1.3[34] (https://github.com/precimed/mixer). MiXeR estimates the total number of trait-influencing variants with nonzero effects, as well as the proportion of shared variants between disorders regardless of the direction of effect. This method is particularly powerful for detecting complex patterns of polygenic overlap, including scenarios with mixtures of concordant and discordant variant effects. Using GWAS summary statistics, the model decomposed genetic architecture into four components: variants influencing both disorders specific to PMD or PPD individually, and null variants associated with neither condition. The estimated number of unique and shared variants were visualized as Venn diagram. The results of MiXeR only represent the quantity rather than the specific variants.

### Pinpoint specific shared variants: conditional/conjunctional false discovery rate

We firstly implemented conditional false discovery rate (condFDR), by combining the power of two GWASs, to improve the detection of common variants associated with PMD and PPD. CondFDR is an extension of the traditional false discovery rate in the empirical Bayesian framework, allowing for inferences about genetic variations associated with primary disease, provided that they are genetically linked to secondary disease [35, 36]. We performed bidirectional conditional analyses to identify novel loci based on the hypothesis that additional SNPs will be discovered in larger samples: (1) re-ranking PMD associations conditioned on PPD *P*-values (PMD∣PPD, condFDR < 0.01), and (2) reciprocally re-ranking PPD associations conditioned on PMD *P*-values (PPD∣PMD, condFDR < 0.01). Before fitting the FDR model, we excluded SNPs around the major histocompatibility complex (MHC) region (chr6:25,119,106–33,854,733, hg19) and chromosome 8 inversion (chr8:7200000–12500000, hg19) to reduce bias, consistent with prior studies [37, 38]. Statistical significance thresholds were maintained at condFDR < 0.01. All analyses were implemented using the R (version 4.3.1) package cfdr.pleio (version 0.0.0.9100) available online (https://github.com/alexploner/cfdr.pleio) with iteration 500 (n_iter = 500).

### Cross-trait meta-analysis of PMD and PPD GWAS

To further increase power, we conducted a fixed-effects inverse-variance weighted meta-analysis using METAL [39], leveraging the largest PMD and PPD GWAS samples currently available. The GWAS summary statistics from the PMD and PPD (excluding MoBa cohort) GWAS datasets. The analysis was performed using the *SCHEME STDERR* option of METAL. Heterogeneity statistics were produced using Cochran’s Q-test. We defined novel loci as those that were significant in the cross-phenotype meta-analysis but not significant in either PMD or PPD input for GWAS.

### Genomic loci definition and functional analysis

Genomic loci were defined using FUMA v1.8.0 (https://fuma.ctglab.nl/) [40]. *P*-value were derived from the METAL meta-analysis results [39]. We identified independent significant SNPs as those reaching a genome-wide significance (METAL *P*-value < 5×10^−8^) with an LD *r*^2^ < 0.6. To capture additional independent signals, we defined candidate SNPs as those with a METAL *P*-value < 0.05 (or reaching a suggestive threshold of 10^−5^) that were in LD (*r*^2^ ≥ 0.6) with the independent significant SNPs. Lead SNPs were further identified using a clumping threshold of *r*^2^ < 0.1. Finally, LD blocks within a 250 kb window were merged into a single genomic locus. LD was calculated using the European ancestry reference panel from the 1000 Genomes Project (phase 3) [41].

Functional annotation and gene mapping were performed for all candidate SNPs using FUMA v1.8.0 (https://fuma.ctglab.nl/) [40], including ANNOVAR, Combined Annotation Dependent Depletion (CADD) scores for predicting the deleteriousness of SNPs on the proteins, RegulomeDB scores for assessing regulatory potentials, and chromatin states to infer transcription/regulatory effects. Gene mapping was conducted using positional mapping (ANNOVAR, hg19), expression quantitative trait locus (eQTL) mapping, and chromatin interaction mapping approaches.

To further characterize the biological relevance of mapped genes, tissue specificity and gene set enrichment analyses were performed using the GENE2FUNC module in FUMA, based on GTEx v8 data and BrainSpan RNA-sequencing data. Gene expression values were log_2_-transformed and normalized to zero mean (TPM for GTEx v8 and RPKM for BrainSpan). Differentially expressed gene (DEG) sets were defined for each tissue or developmental stage using two-sided Student’s t tests comparing each label against all remaining labels. Genes with Bonferroni-corrected *P* ≤ 0.05 and an absolute log fold change ≥ 0.58 were defined as DEGs. Upregulated and downregulated DEG sets were further defined based on the sign of the t statistics. Genes mapped by all three mapping strategies were tested for enrichment in each DEG set using the hypergeometric test, with background genes defined as those with an average expression value > 1 in at least one label and present in the user-defined background gene set. Bonferroni correction was applied separately for upregulated, downregulated, and combined DEG sets.

We then selected GWAS mapped genes to conduct global pathway enrichment analysis using the online software Metascape (http://metascape.org/) [42]. Results were automatically generated by Metascape using default parameters. Gene annotations automatically retrieved from the latest version of the database are shown. All genes in the genome were used as the enrichment background. Terms with *P* < 0.01, a minimum count of 3, and an enrichment factor > 1.5 were collected and grouped into clusters based on their similarities. The enrichment factor is the ratio of the observed counts and the counts expected by chance. The top 9 clusters with their representative enriched terms (one per cluster) are provided here. *P* values were calculated based on the cumulative hypergeometric distribution. Log10(q), the multiple-testing-adjusted *P* value in log base 10, was calculated using the Benjamini–Hochberg procedure. Pathways with a multiple-testing-adjusted *P* < 0.05 are usually considered statistically enriched.

### Transcriptome-wide association analyses (TWAS)

TWAS were conducted using complementary tissue-specific and cross-tissue analytical frameworks to evaluate gene expression–mediated genetic associations for PMD and PPD. Tissue-specific TWAS analyses were first performed using FUSION, which integrates GWAS summary statistics with expression quantitative trait loci (eQTL) reference panels to test for associations between genetically predicted gene expression and disease risk [43]. Gene expression weights were derived using multiple predictive models implemented in FUSION, and tissue-specific association statistics were obtained across all available GTExv8 tissues (49 tissues from the ALL and EUR populations). In addition to single-tissue models, analyses also included cross-tissue sparse canonical correlation analysis (sCCA) components and pre-computed expression reference panels for peripheral blood and whole blood provided by FUSION website (http://gusevlab.org/projects/fusion/).

### Meta-analysis of baseline lymphoblastoid cell line (LCLs) transcriptomes

To replicate lead markers from our genetic analyses, we restricted the LCL transcriptomic meta-analysis to a predefined set of 702 genes (213 GWAS-mapped genes and 524 TWAS Trend/Sig genes, with 35 overlapping genes). We performed meta-analysis using human derived baseline LCL differential expression results from two published studies [44, 45]. These studies used Epstein Barr virus (EBV)-transformed LCLs [46] derived from peripheral blood lymphocytes, and only the untreated/baseline condition from each study was included in the present analysis [44, 45]. In the PMDD study, the untreated LCLs data were derived from a hormonally validated case-control sample, including women with clinical confirmed PMDD whose symptom remission and recurrence were confirmed during GnRH-agonist ovarian suppression and estradiol/progesterone add-back, together with asymptomatic controls [44]. In the PPD study, the baseline LCL data were derived from a strictly phenotyped, well-matched case-control sample of clinically characterized parous women with a history of PPD and matched controls [45]. PMDD statistics were taken from the PMDD LCL study, using the supplementary Control-vs-PMDD gene-level table (Table OL1.xlsx) [44]. PPD statistics were taken from the PPD LCL study, using Supplementary Table 1 (file NIHMS1925041-supplement-Sup_Table_1.xls, sheet PPD_DGE_Baseline_SupTable1) [45]. After harmonizing gene symbols, 286/702 genes (40.7%) were available in both datasets. Because PMDD and PPD effect sizes were on different scales (fold change vs logFC), we used a p-value-based signed Stouffer meta-analysis with direction defined by the sign of fold change/logFC. Concordant signals were defined as same-direction genes with signed Stouffer MetaP < 0.05, yielding 22 genes.

## Results

### Genetic correlation and shared polygenic liability between PMD and PPD

In this nationwide cohort of all women who were born during 1950-2007, and gave birth during 2001-2021 in Sweden, we identified 907,841 parous women and 1,056,387 full sister pairs (**Table S1**). We observed a prevalence of 9.16% (95% CI: 9.08-9.24%) for PMD and 8.64% (95% CI: 8.58-8.70%) for PPD in this population. The CIF-based heritability was estimated as 0.35 (95% CI: 0.29-0.41) for PMD and 0.31 for PPD (95% CI: 0.23-0.37). A positive genetic correlation between PMD and PPD was observed (*r*_2_ = 0.47, 95% CI: 0.25–0.69), supporting shared genetic liability at the population level.

**Figure 1.**
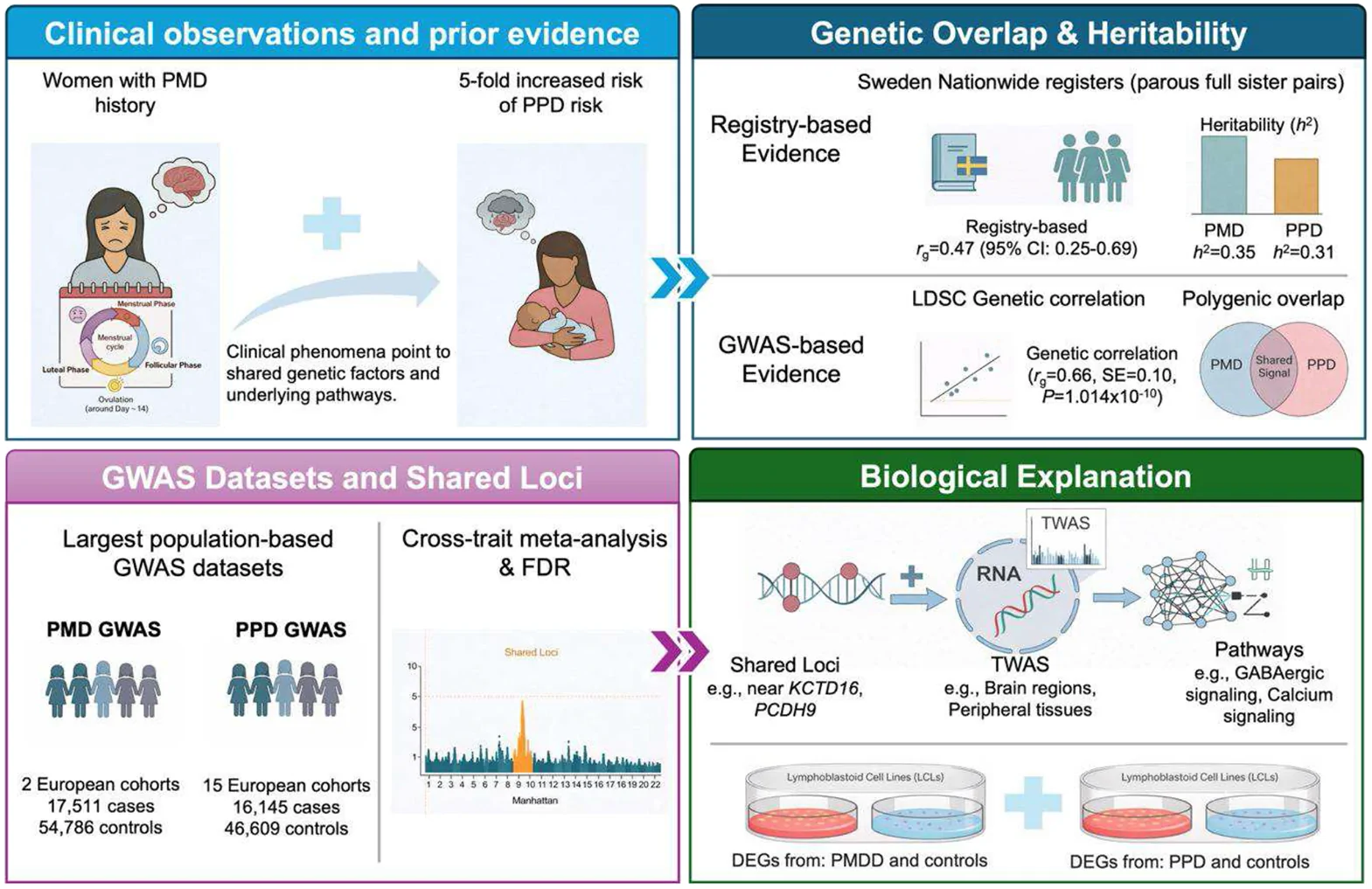
Study design and analytical framework. The figure outlines the rationale, datasets, and analytical approach. Genetic overlap and heritability were assessed using GWAS-based approaches, including LDSC and MiXeR, and further evaluated using Swedish nationwide registry data. The GWAS analyses included a PMD dataset comprising 17,511 cases and 54,786 controls from 2 European cohorts and a PPD dataset comprising 16,145 cases and 46,609 controls from 15 European cohorts. Shared loci and biological pathways were identified using cross-trait meta-analysis, condFDR, transcriptome-wide association analysis, and pathway enrichment analyses.

To quantify the extent of shared genome-wide genetic liability between PMD and PPD, as shown in Figure 1, we utilized the largest PMD GWAS (17,511 cases and 54,786 controls of European ancestry from two cohorts) and independent PPD GWAS (16,145 PPD cases and 46,609 controls of European ancestry from 15 cohorts).

After harmonizing and filtering of shared SNPs, we applied linkage disequilibrium score regression (LDSC) to estimate genome-wide genetic correlation (*r*_g_) between PMD and PPD [31, 32]. The two disorders showed a significant positive genetic correlation (*r*_2_ = 0.66, SE = 0.10, *P* = 1.014×10^−10^), supporting the presence of shared genetic liability between PMD and PPD. We then applied MiXeR v1.3 [34] to characterize their polygenic architecture. The univariate MiXeR model demonstrated adequate fit for PMD (AIC = 4.23; BIC = 14.71), consistent with a highly polygenic genetic architecture, with an estimated 7,701 variants among the modeled SNPs. The univariate MiXeR analysis of PPD GWAS showed poor model fit (AIC = −28.31; BIC = −17.46), precluding estimation of trait-influencing variant counts and subsequent bivariate MiXeR analysis.

### Identification of shared risk loci between PMD and PPD

To identify specific variants contributing to shared genetic risk, we applied the conditional false discovery rate (condFDR) framework, which leverages association strength in one trait to improve discovery in a genetically related second trait [35, 36]. Using a condFDR threshold of 0.01, we identified multiple loci exhibiting strengthened evidence of association when conditioning on the alternate phenotype (PMD∣PPD or PPD∣PMD). These loci included *CACNA1C*, *KCTD16*, *KCNG2*, *SMYD1*, and additional regions which did not reach genome-wide significance in single-trait GWAS (*P* < 5 × 10^−8^) but demonstrated consistent cross-trait enrichment (**Table S2**). These findings indicate that a subset of shared genetic signals between PMD and PPD remains distributed below standard genome-wide thresholds and can be uncovered by leveraging cross-disorder information, consistent with a shared polygenic architecture.

**Figure 2.**
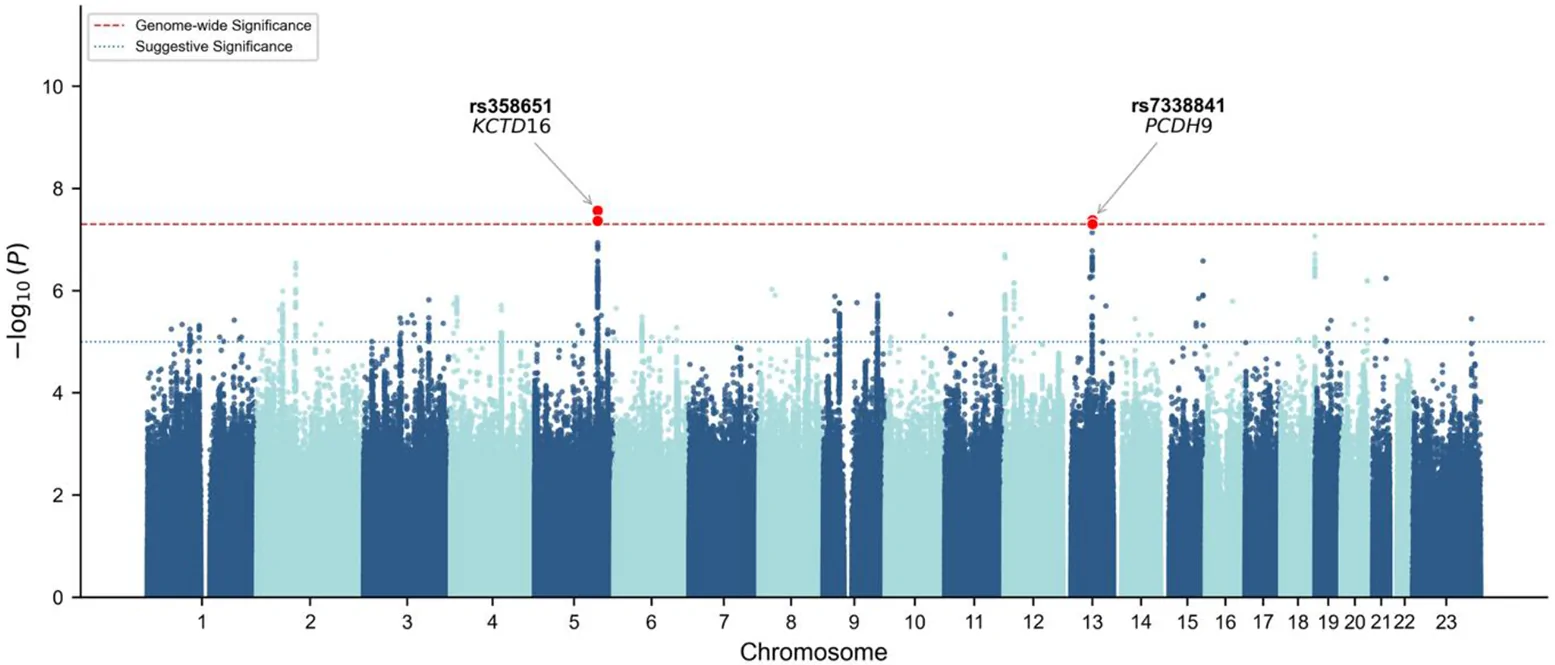
Manhattan plot of the meta-analyzed GWAS of PMD and PPD. We conducted a cross-phenotype genome-wide association meta-analysis of PMD and PPD using a fixed-effect model. The Manhattan plot displays the −log_10_(P) values of SNP associations across genome. The red dashed line indicates the genome-wide significance threshold (*P* < 5 × 10^−8^), and the blue dotted line indicates the suggestive significance threshold (*P* < 1 × 10^−5^). Five genome-wide significant loci are labeled.

To increase statistical power for locus discovery, we then conducted a fixed-effect inverse-variance weighted meta-analysis using METAL [39, 47], integrating the PMD and PPD GWAS summary statistics. The meta-analysis showed modest inflation (genomic inflation factors (λ_GC_) = 1.130), while the LDSC intercept remained close to 1 (1.0075, SE = 0.0057), supporting polygenicity rather than substantial confounding. The quantile–quantile (Q–Q) plot indicated deviation from the null only at the extreme tail (**Figure S1**). A total of 562 variants reached suggestive significance (*P* < 1 × 10^−5^, **Table S3**), among which five SNPs exceeded the genome-wide significance threshold (*P* < 5 × 10^−8^) (Figure 2; Table 1). These five SNPs clustered into two independent loci (rs358651, rs7338841), each exhibiting consistent direction of effect in PMD and PPD, supporting their role in shared genetic susceptibility.

To evaluate whether the meta-analytic findings were supported by signals in each disorder, we examined regional association patterns for these two lead loci using LocusZoom with the hg19/1000 Genomes Nov 2014 European LD reference and a ±500 kb window around each index SNP (**Figure S2**). At the chromosome 5 locus, rs358651 within the 3’ untranslated region (UTR) of *KCTD16*, and both displayed a single, well-defined LD peak without evidence of secondary association signals, with a stronger association signal for PMD compared with PPD (**Figure S2A, B**). At the chromosome 13 locus, rs7338841 within intronic region of *PCDH9* exhibited highly similar regional association profiles between PMD and PPD, with comparable signal strength, supporting a shared association across PMD and PPD (**Figure S2C, D**). Together, these meta-analysis results indicate two lead loci with consistent associations shared between PMD and PPD, although their strength of association differs.

### Genome-wide significant loci implicate GABAergic and calcium signals

The top locus was rs358651 (β_meta_ = −0.056, *P_meta_* = 2.72 × 10^−8^) on chromosome 5, which maps to the 3’ UTR of *KCTD16*, based on positional annotation using ANNOVAR (Table 1). *KCTD16* encodes an auxiliary subunit of the GABA_B_ receptor complex and, together with other KCTD family members (KCTD8, KCTD12), plays a central role in regulating the kinetics and strength of inhibitory GABA_B_ signaling through modulation of G protein–coupled inwardly rectifying potassium (GIRK) channels, thereby shaping inhibitory neurotransmission [48].

The other lead locus, rs7338841 (β_meta_ = 0.0509, *P_meta_* = 4.15 × 10^−8^) is an intronic variant within *PCDH9* on chromosome 13, as annotated by ANNOVAR in Table 1. *PCDH9* encodes a protocadherin-family transmembrane adhesion molecule highly expressed in the brain. PCDH9 participates in calcium-dependent neuronal adhesion, synaptic connectivity and circuit formation, processes essential for neural network stability and affective behaviors.

We queried the GWAS Catalog to assess whether the lead loci had been previously implicated in human traits, rs358651 located at the 3’ UTR of *KCTD16*, has not been reported in prior GWAS for psychiatric or reproductive mood phenotypes. For rs7338841, several SNPs in high LD within the same region have been associated with neuroticism, insomnia, and cigarette consumption, predominantly in large European-ancestry cohorts, as well as with vaginal microbiome composition (**Table S4**).

Functional annotation to characterize loci defined at the suggestive significance threshold (*P* < 1 × 10^−5^) was then performed. Using FUMA SNP2GENE [40], we identified 80 genomic risk loci, corresponding to 85 lead SNPs, 97 independent significant SNPs (**Figure S3A**). Gene mapping using three strategies, positional mapping (hg19), eQTL mapping, and chromatin interaction mapping, mapped these loci to 213 mapped genes. Candidate SNPs were predominantly located in non-coding regions, particularly intergenic and intronic regions, with few mapping to coding sequences (**Figure S3B**). Developmental expression analysis using BrainSpan showed limited overall enrichment, with nominal signals at 21 and 24 post-conception weeks (pcw) (**Figure S3C**). Gene-set and pathway enrichment analysis for these 213 genes using Metascape (http://metascape.org/) [42] demonstrated broad enrichment of cell–cell adhesion–related processes (**Figure S3D**).

### Transcriptome-wide analyses suggest tissue-specific mediation of shared risk

**Figure 3.**
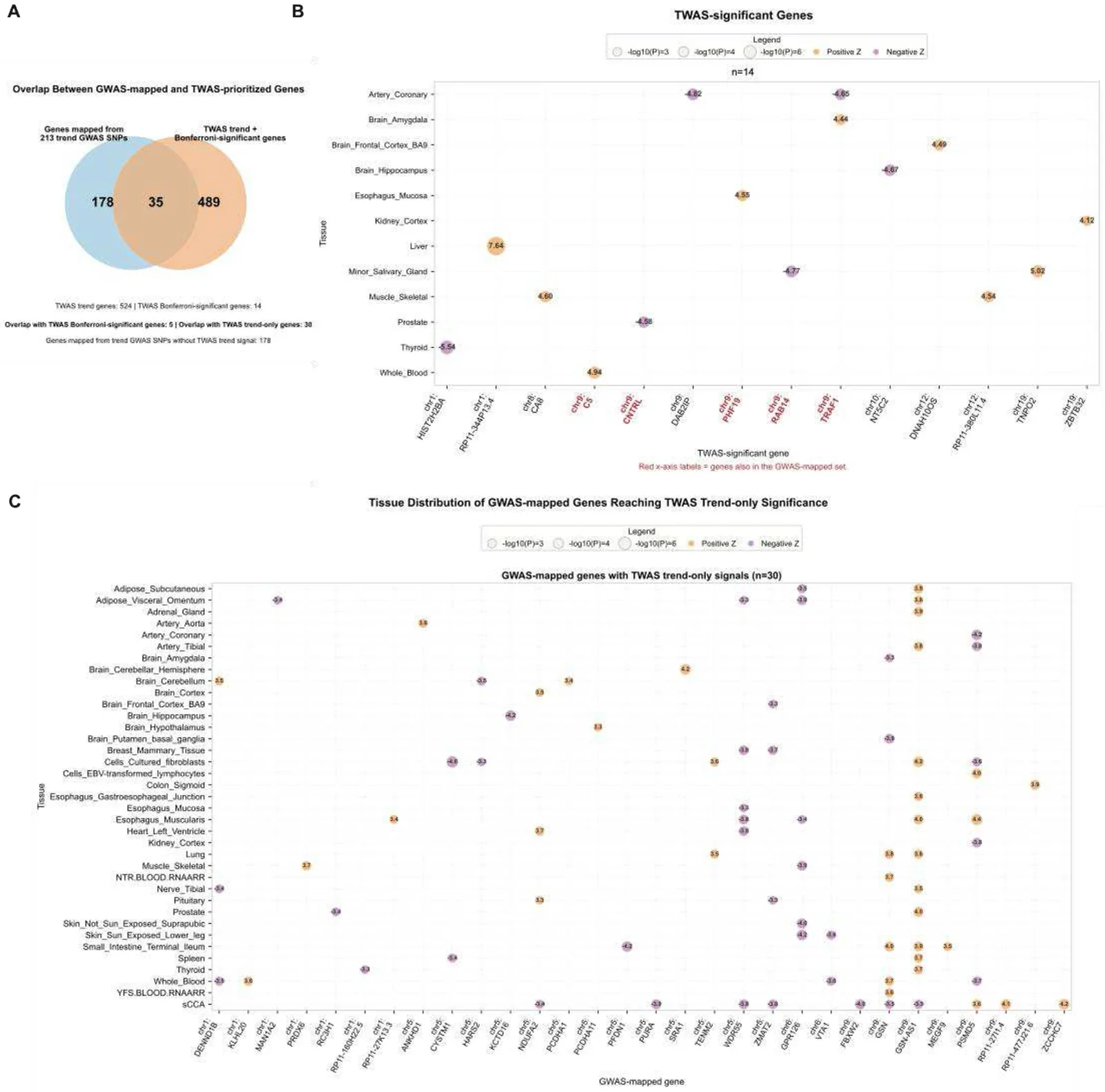
GWAS-mapped and TWAS-prioritized genes highlight shared candidate genes across tissues. A. Overlap between GWAS-mapped genes and TWAS-prioritized genes (TWAS.*P* < 0.001). B. Significant gene–tissue associations identified by TWAS. Genes shown in red on the x axis overlapped between the GWAS-mapped and TWAS-significant gene sets. C. Tissue distribution of GWAS-mapped genes reaching TWAS threshold significance (TWAS.*P* < 0.001). Each bubble represents a gene–tissue pair; bubble size is proportional to −log_10_(*P*), and bubble color indicates the direction of the TWAS Z-score (orange, positive; purple, negative). For each gene–tissue pair, the most significant association across available pre-trained models is shown.

We performed TWAS analysis to evaluate gene-expression–mediated associations for PMD and PPD leveraging METAL results. Available trained expression prediction models for FUSION include GTEx v8 tissues (49 tissues of ALL and EUR populations), cross-tissue spare canonical correlation analysis (sCCA) components, and pre-computed expression reference (peripheral blood and whole blood) with reliable expression prediction models (http://gusevlab.org/projects/fusion/). The full TWAS results across these expression reference panels and tissues were merged into **Table S5** for details. 35 genes were overlapped between GWAS-mapped and TWAS prioritized genes (TWAS.*P* < 0.001) (Figure 3A). Among TWAS prioritized genes, we identified several (N = 14) significant gene-tissue associations after applying Bonferroni correction (0.05/N_genes_ in each model) (Figure 3B), including *HIST2H2BA*, *CA8*, *C5*, *DAB2IP*, *PHF19*, *RAB14*, *TRAF1*, *NT5C2*, and *ZBTB32*. These associations originated from different tissues, including whole blood, multiple brain regions (e.g., hippocampus, amygdala, frontal cortex) and endocrine and other tissues such as thyroid and esophageal mucosa.

To complement these transcriptome-wide findings, we then assessed whether genome-wide significant and suggestive METAL SNPs (*P* < 1 × 10^−5^) showed evidence for expression-mediated effects. These variants were mapped to nearby or functionally plausible genes, and a suggestive threshold (TWAS *P* < 1 × 10^−3^) was applied to identify associations. We plotted gene-tissue associations in each tissue for these positional mapped genes (Figure 3C). The transcriptional architecture at the two genes mapped from the independent lead SNPs (*P* < 5 × 10^−8^: rs358651, located on chromosome 5 within the 3’ untranslated region of *KCTD16*; rs7338841, an intronic variant in *PCDH9* on chromosome 13) differed. For *KCTD16*, TWAS detected a tissue association in the hippocampus, with a negative Z-score, indicating that higher genetically predicted expression may be associated with lower PMD-PPD risk. No other GTEx v8 tissues (in European population) or sCCA components reached *P* < 1 × 10^−3^ for this gene. By contrast, no GTEx EUR/ALL tissue or sCCA component showed a TWAS association at *P* < 0.001 for rs7338841 at the *PCDH9* locus. The absence of detectable expression-mediated effects reflects that incomplete representation of this locus in current FUSION expression prediction models, which are based on cis-SNP expression weights rather than all variants across the locus.

### Lead GWAS genes present in lymphoblastoid cell line (LCLs) transcriptomes

To replicate genes mapped by leading markers identified in our genetic analyses, we restricted transcriptomic follow-up to the 702 predefined GWAS/TWAS-prioritized genes (Figure 3A, 213 GWAS-mapped genes and 524 TWAS trend/significant genes, with 35 overlapping genes). Using baseline (i.e., ovarian steroid-free) LCL differential-expression results (empirical *P*<0.05) from published PMDD (prospectively diagnosed cases) [44] and PPD studies [45], we found that 286 of these 702 genes (40.7%) were present in both datasets after harmonization of gene symbols. Many GWAS-prioritized genes (e.g., *KCTD16* and *PCDH9*) were not expressed in LCLs. Among the 286 overlapping genes, 155 (54.2%) showed concordant directions of effect in both datasets. Of these, 22 had signed Stouffer Meta.*P* < 0.05, including 8 GWAS-mapped genes and 17 TWAS trend/significant genes, with *PFDN1*, *C5*, and *GSN* supported by both approaches (**Table S6**).

## Discussion

This study investigated the shared genetic architecture between PMD and PPD using Swedish nationwide population register data and family-based analyses, together with the largest large-scale GWAS summary statistics and cross-trait genomic and functional integrative approaches. These complementary analyses provided convergent evidence for substantial sizable shared heritable liability between PMD and PPD, despite these conditions manifesting at two distinct hormonally dynamic periods of female reproductive life course.

At both the population- and genome-wide levels, the observed positive and largely comparable genetic correlation supports overlapping common variant between PMD and PPD. Polygenic modelling revealed that PMD exhibits a highly polygenic architecture, whereas the polygenicity of PPD could not be stably estimated from the current GWAS. This may reflect limited power, greater heterogeneity in PPD case definitions across cohorts, or both. Collectively, these findings suggest that shared genetic liability is distributed across many variants with mixed directional effects, such that global correlation metrics alone may underestimate the extent of cross-disorder genetic overlap, consistent with prior cross-trait psychiatric studies [34]. To better characterize this shared architecture, we used fixed-effect cross-trait meta-analysis and conditional FDR–based enrichment. The fixed-effect meta-analysis identified 2 novel genome-wide significant loci with concordant allelic effects across PMD and PPD, including rs358651 mapping to the 3’ UTR of *KCTD16* and rs7338841 mapping to the intronic region within *PCDH9*. In addition, the condFDR analyses highlighted additional loci showing strengthened evidence when leveraging cross-disorder enrichment, consistent with a polygenic architecture in which many shared signals remain below genome-wide thresholds in single-trait analyses [35].

To date, the lead variant rs358651 located at the 3’ UTR of *KCTD16* has not been reported in prior GWAS for psychiatric or reproductive mood phenotypes, highlighting this region as a novel shared locus. Existing studies on PMD/PPD and the only FDA approved medication for the treatment of PPD have primarily emphasized the importance of GABAergic inhibitory signaling in mood regulation and stress sensitivity, particularly emphasizing neurosteroid-sensitive modulation of GABA_A_ receptors [49-51]. However, the role of other GABA receptors is less studied. *KCTD16* encodes an auxiliary subunit of the GABA_B_ receptor complex and together with *KCTD8*, *KCTD12*, plays a central role in regulating the kinetics and strength of inhibitory GABA_B_ signaling through modulation of G protein–coupled inwardly rectifying potassium (GIRK) channels, thereby shaping inhibitory neurotransmission [48]. High-resolution structural studies further demonstrate how KCTD16 interacts with the GABA_B2_ subunit to fine-tune the temporal properties of receptor signaling [48, 52]. A mouse study *in situ* hybridization showed that widespread brain expression of *Kctd16* transcripts and further suggested that individual neurons in the hippocampus, including granule cells and CA1/CA3 pyramidal neurons, could co-express KCTD12 and KCTD16 subunits [53]. Our TWAS results identified a localized hippocampus regulatory signal for *KCTD16*, suggesting that genetically predicted expression of this gene may modulate shared PMD–PPD risk through hippocampal inhibitory circuitry. Together, our findings point to GABA_B_-dependent inhibitory signaling as an additional and previously underexplored component of susceptibility to hormonally sensitive mood dysregulation.

The other lead variant rs7338841 maps to the intronic region within *PCDH9*. Several SNPs in high LD within the same region have been associated with neuroticism, insomnia, and cigarette consumption, predominantly from large European-ancestry cohorts, as well as with vaginal microbiome composition, as reported in the GWAS Catalog (**Table S3**). *PCDH9* encodes a protocadherin-family transmembrane adhesion molecule highly expressed in the brain, which participates in calcium-dependent neuronal adhesion, synaptic connectivity and circuit formation, processes essential for neural network stability and affective behaviors. One experimental study reported that disruption of *Pcdh9* affects social behavior and cortical development, supporting a potential role in neurobehavioral phenotypes [54]. *PCDH9* has been implicated in major depressive disorder (MDD) with convergent evidence from GWAS and gene expression analyses [55]. Notably, *PCDH9* did not exhibit detectable expression-mediated effects in current GTEx analyses, suggesting that risk at this locus may act through non-expression-based regulatory processes not captured by adult bulk tissue eQTL resources, a pattern commonly observed in neuropsychiatric genetics. Global TWAS analysis suggests that shared PMD–PPD genetic variants are conveyed through heterogeneous yet convergent expression-mediated mechanisms spanning brain regions (such as amygdala, hippocampus) [56], endocrine and immune systems [57], rather than a single tissue as shown in Figure 3.

Exploratory cross-level convergence between genetic and transcriptomic signals highlighted three prioritized candidate *PFDN1*, *C5* and *GSN*. *PFDN1* was prioritized as a possible causal gene in shared genomic risk regions from a sex-stratified GWAS meta-analysis of MDD [58]. *C5* provides convergent support for immune involvement. Cerebrospinal fluid C5 levels were reported to be increased in patients with MDD [59]. Higher plasma C5 levels in childhood have been associated with later psychotic experiences in a longitudinal cohort, suggesting broader psychiatric vulnerability rather than an MDD-specific effect [60]. Finally, exploratory proteomic studies have implicated *GSN* (gelsolin) in depression-related protein signatures [61]. Together, these findings suggest potential involvement of proteostasis and immune regulation in reproductive mood disorders. However, the overlap should be interpreted cautiously, as LCLs are an in vitro peripheral surrogate model that captures only part of the relevant *in vivo* biology [62]. Moreover, many GWAS-prioritized genes (e.g., *KCTD16* and *PCDH9*) were not expressed in LCLs or may be expressed but may not necessarily act through detectable baseline expression differences.

A major strength of this study lies in using different datasets and approaches to investigate the shared genetic architecture between PMD and PPD. PMD is characterized by symptom onset tightly linked to menstrual cycle-related hormonal fluctuations, and case ascertainment in our Nordic cohorts was explicitly anchored on clinical diagnoses and questionnaire-based assessments. This phenotypic specificity provides a strong framework for investigating genetic liability to hormonally sensitive mood dysregulation. Examining PMD alongside PPD also allowed us to assess whether shared genetic liability extends across mood disorders occurring at different hormonally sensitive stages of the reproductive life course.

Several limitations also should be acknowledged. PPD represents a more heterogeneous clinical entity, with variation in symptom onset, severity, and ascertainment across cohorts. Importantly, not all PPD cases are driven primarily by hormone-related mechanisms [63]. Against this background, the identification of shared genetic signals between PMD and PPD suggests that a biologically defined subset of PPD characterized by heightened sensitivity to hormone changes may otherwise be diluted by studying PPD as one group. In addition, case definitions partly relied on screening instruments and registry diagnoses, which introduce some degree of misclassification. However, such misclassification is expected to be largely non-differential with respect to genetic variants and would therefore tend to attenuate rather than inflate associations. Although prospective symptom charting and direct psychiatric interviews represents the diagnostic gold standards, neither is feasible in samples of the size required for adequately powered GWAS and may introduce substantial loss to follow-up when administering at the cohort level [64]. The absence of validated biological markers reflects limited mechanistic understanding of the disorder, which constrains diagnostic precision across the field. Large-scale genetic studies such as ours are therefore essential to provide biological insights and if confirmed later in prospective charting-diagnosed cases, can improve future classification. Future studies incorporating explicit stratification by psychiatric comorbidities and prior non-reproductive mood disorder history are needed to clarify the extent to which the observed shared liability reflects PMD-PPD-specific overlap versus broader psychiatric genetic risk. The accuracy of genetically predicted expression is constrained by expression heritability, and current models primarily capture cis-regulatory effects, while trans-regulatory and context-specific mechanisms are not evaluated [43, 65]. In addition, TWAS is inherently association-based and does not establish causality [66], underscoring the need for complementary functional validation. Finally, all analyses were restricted to individuals of European ancestry, and the generalizability of these findings to other populations remains to be established.

## Conclusions

In summary, our results provide the first genome-wide evidence on a sizable genetic overlap between PMD and PPD. The identification of novel loci shared between PMD and PPD, for instance the locus in *KCTD16*, highlights GABA_B_-mediated inhibitory signaling as potential underlying component. More broadly, the convergence of neural, endocrine and immune pathways supports a multi-system model in which shared genetic risk manifests through heterogeneous yet interconnected biological mechanisms underlying the affective susceptibility to hormone fluctuations across the female reproductive lifespan.

## Supplementary Material

This is a list of supplementary files associated with this preprint. Click to download.

- PMDPPDgeneticoverlapSuppFigures260509.docx
- PMDPPDgeneticoverlapSuppTables260509.xlsx

## Figures and Tables

**Table 1.** GWAS meta-analyses revealed genome-wide significant SNPs (*P* <5×10^−8^).

							Meta-analysis				PMD		PPD	
SNP	CHR	BP	Allele1	Allele2	Annot	Nearest Gene	Beta	SE	*P*-value	P for heterogeneity	Beta	*P*-value	Beta	*P*-value
rs358651 (lead SNP)	5	143855606	a	g	UTR3	*KCTD16*	−0.056	0.010	2.72E-08	0.172	−0.068	3.58E-07	−0.040	8.62E-03
rs358673	5	143881073	t	g	intergenic	*KCTD16*	0.055	0.010	4.27E-08	0.246	0.066	9.64E-07	0.042	6.32E-03
rs7338841 (lead SNP)	13	66994425	a	g	intronic	*PCDH9*	0.051	0.009	4.15E-08	0.987	0.051	5.44E-05	0.051	2.19E-04
rs9540745	13	66992172	c	g	intronic	*PCDH9*	0.051	0.009	4.29E-08	0.991	0.051	5.64E-05	0.051	2.17E-04
rs4884671	13	66995749	a	g	intronic	*PCDH9*	0.051	0.009	4.98E-08	0.987	0.051	6.03E-05	0.050	2.37E-04

Note:

PMD = Premenstrual disorder, PPD = Postpartum depression, SNP = single nucleotide polymorphism, CHR= chromosome, BP= Position on the chromosome (GRCh37 genomic build), Allele 1= Risk allele, A2= reference allele, Annot is annotated from ANNOVAR.

## List of abbreviations

AIC: Akaike information criterion
ATC: Anatomical Therapeutic Chemical
BIC: Bayesian information criterion
CADD: Combined Annotation Dependent Depletion
CI: Confidence interval
CIF: Cumulative incidence function
condFDR: Conditional false discovery rate
DEG: Differentially expressed gene
DSM: Diagnostic and Statistical Manual of Mental Disorders
EBV: Epstein–Barr virus
EPDS: Edinburgh Postnatal Depression Scale
eQTL: Expression quantitative trait locus
EUR: European ancestry
FDR: False discovery rate
GABA: Gamma-aminobutyric acid
GIRK: G protein-coupled inwardly rectifying potassium
GTEx: Genotype-Tissue Expression
GWAS: Genome-wide association study
ICD: International Classification of Diseases
LCL: Lymphoblastoid cell line
LD: Linkage disequilibrium
LDSC: Linkage disequilibrium score regression
MAF: Minor allele frequency
MDD: Major depressive disorder
MGR: Multi-Generation Register
MHC: Major histocompatibility complex
MoBa: Norwegian Mother, Father and Child Cohort Study
NPR: National Patient Register
PCR: Primary Care Register
PDR: Prescribed Drug Register
PMD: Premenstrual disorder
PMDD: Premenstrual dysphoric disorder
PPD: Postpartum depression
PSST: Premenstrual Symptoms Screening Tool
RPKM: Reads per kilobase of transcript per million mapped reads
sCCA: Sparse canonical correlation analysis
SE: Standard error
SNP: Single nucleotide polymorphism
TPR: Total Population Register
TPM: Transcripts per million
TWAS: Transcriptome-wide association study
UTR: Untranslated region
